# Panobinostat mediated cell death: a novel therapeutic approach for osteosarcoma

**DOI:** 10.18632/oncotarget.26038

**Published:** 2018-08-31

**Authors:** André Wirries, Samir Jabari, Esther P. Jansen, Silvia Roth, Elizabeth Figueroa-Juárez, Thaddeus T. Wissniowski, Daniel Neureiter, Eckhard Klieser, Philipp Lechler, Steffen Ruchholtz, Detlef K. Bartsch, Christoph K. Boese, Pietro Di Fazio

**Affiliations:** ^1^ Center of Orthopaedics and Trauma Surgery, Philipps University of Marburg, Baldingerstrasse 35043 Marburg, Germany; ^2^ Institute of Anatomy I, University of Erlangen-Nuremberg, 91054 Erlangen, Germany; ^3^ Department of Visceral, Thoracic and Vascular Surgery, Philipps University of Marburg, Baldingerstrasse 35043 Marburg, Germany; ^4^ Department of Gastroenterology and Endocrinology, Philipps University of Marburg, Baldingerstrasse 35043 Marburg, Germany; ^5^ Institute of Pathology, Paracelsus Medical University/Salzburger Landeskliniken (SALK), 5020 Salzburg, Austria; ^6^ Salzburg Cancer Research Institute, 5020 Salzburg, Austria; ^7^ Department of Orthopaedic and Trauma Surgery, University Hospital of Cologne, 50937 Cologne, Germany; ^8^ Orthopaedic Clinics, Hessing Foundation, 86199 Augsburg, Germany

**Keywords:** osteosarcoma, endoplasmic reticulum stress, autophagy, histone deacetylase inhibitor, cell death

## Abstract

Osteosarcoma is an aggressive cancer with a poor long term prognosis. Neo-adjuvant poly-chemotherapy followed by surgical resection remains the standard treatment, which is restricted by multi-drug resistance. If first-line therapy fails, disease control and patient survival rate drop dramatically. We aimed to identify alternative apoptotic mechanisms induced by the histone deacetylase inhibitor panobinostat in osteosarcoma cells.

Saos-2, MG63 and U2-OS osteosarcoma cell lines, the immortalized human osteoblast line hFOB and the mouse embryo osteoblasts (MC3T3-E1) were treated with panobinostat. Real time viability and FACS confirmed the cytotoxicity of panobinostat. Cell stress/death related factors were analysed by RT-qPCR and western blot. Cell morphology was assessed by electron microscopy.

10 nM panobinostat caused cell viability arrest and death in all osteosarcoma and osteoblast cells. P21 up-regulation was observed in osteosarcoma cells, while over-expression of p73 was restricted to Saos-2 (TP53^−/−^).

Survivin and Bcl-2 were suppressed by panobinostat. Endoplasmic reticulum (ER) stress markers BiP, CHOP, ATF4 and ATF6 were induced in osteosarcoma cells. The un-spliced Xbp was no further detectable after treatment.

Autophagy players Beclin1, Map1LC3B and UVRAG transcripts over-expressed after 6 hours. Protein levels of Beclin1, Map1LC3B and p62 were up-regulated at 72 hours. DRAM1 was stable. Electron micrographs revealed the fragmentation and the disappearance of the ER and the statistically significant increase of autophagosome vesiculation after treatment.

Panobinostat showed a synergistic suppression of survival and promotion of cell death in osteosarcoma cells. Panobinostat offers new perspectives for the treatment of osteosarcoma and other malignant bone tumours.

## INTRODUCTION

Osteosarcoma is the most frequent primary malignant bone tumour in children and adolescents with a second peak in the elderly [1, 2].

For the past decades, the overall 5-year survival rate has been stagnating at 70–80% in non-metastatic, relapse free cases [3, 4]. The detection of metastasis leads to a 5-year survival rate of 20–30% [2, 5, 6]. Multiple variations in neo-adjuvant poly-chemotherapy did not fulfil expectations and there is still a high demand for more effective drugs [7, 8]. Especially the occurrence of multi drug resistance, the primary cause of poor survival rate in metastatic or relapsed disease, has to be addressed [3, 4, 7, 9, 10].

Alternative approaches include deacetylase inhibitors that are responsible for modulating the epigenetic code of tumour cells. Apoptosis, autophagy and endoplasmic reticulum (ER)-stress respond to these epigenetic modifications and play a major role in the development of chemotherapeutic resistance. The deacetylase inhibitor panobinostat (LBH-589 by Novartis, Basel Switzerland) has been shown to influence these epigenetic pathways [11–17] and therefore represents a valid alternative treatment option in primary malignant bone tumours including osteosarcoma [8, 18].

In particular the ability to tilt and reactivate these fine-tuned cellular fate controller mechanisms could offer a new strategy to block osteosarcoma cell proliferation and survival [8, 19, 20]. Chromatin hypo-acetylation caused by the modification of histone proteins, seems to play a key role during tumorigenesis [21]. Promoting the hyper-acetylation of histone and non-histone proteins by the use of histone deacetylase inhibitors (HDACi), e.g. panobinostat, promotes cell death in several solid cancers and is currently successfully tested in clinical trials for the treatment of hematologic and lymphoid cancers [22].

Deacetylase inhibitors have shown to activate several mechanisms promoting cell demise not only *via* canonical apoptosis but also through the activation of alternative cell death mechanisms like ER stress and autophagy [12, 15, 23].

Autophagy describes the ability of eukaryote cells to degrade cellular molecules, organelles and proteins using autophagosomes as carriers [24]. Normally the induction of autophagy related cell stress is linked to the promotion of cell survival but [25], under certain conditions, elevated autophagy levels lead to cell demise representing an alternative way of cell death [13, 24, 26, 27].

Accumulation of premature proteins in the ER induces a process called unfolded protein response, known to be capable of activating autophagy and therefore being responsible for promoting cell death or survival, respectively [28–30].

We hypothesized that the deacetylase inhibitor panobinostat induces an alternative way of cell death by promoting ER stress mediated autophagy in osteosarcoma cells.

## RESULTS

### Osteosarcoma cell viability

**Figure 1:**
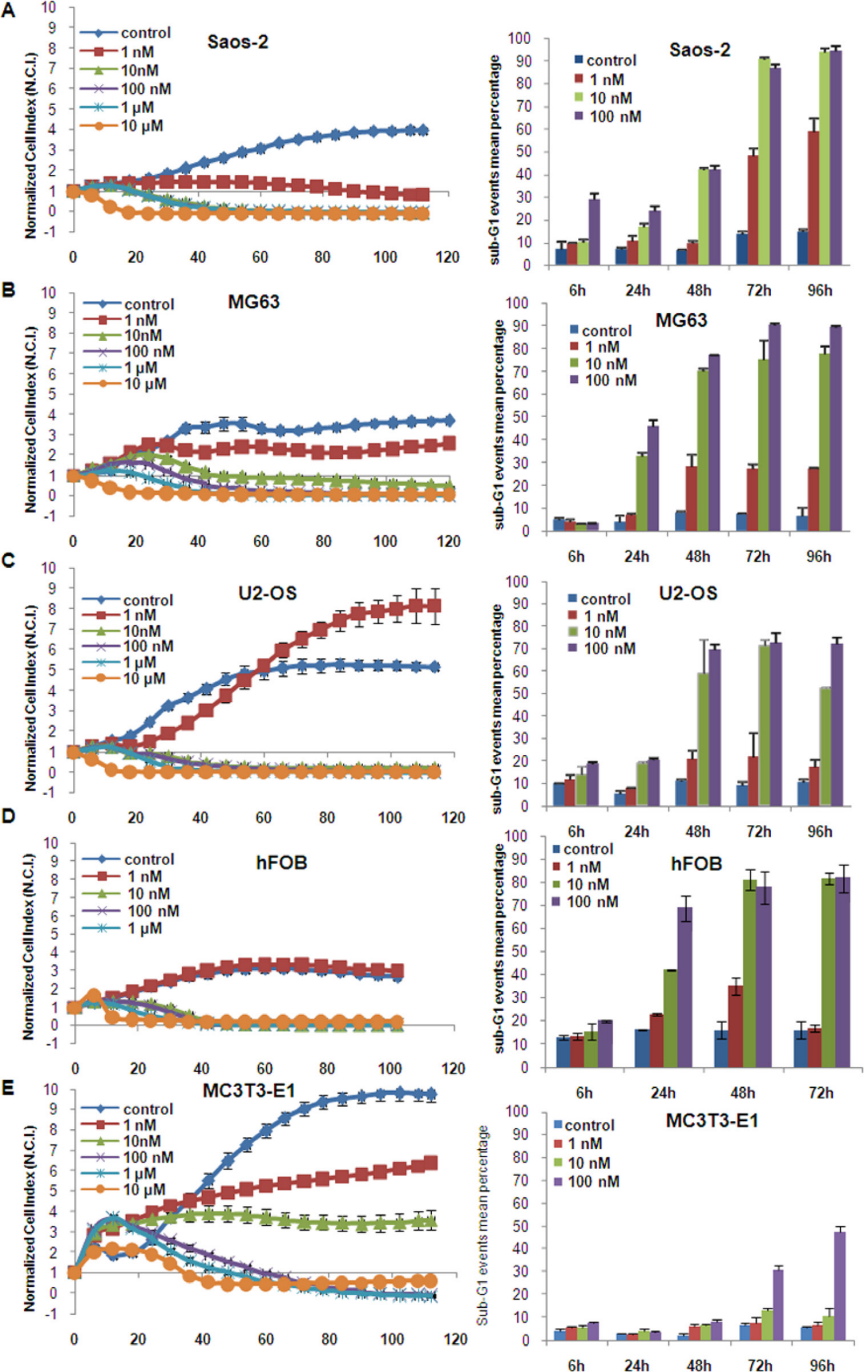
Panobinostat effect on cell viability Saos-2 (**A**), MG63 (**B**), U2-OS (**C**), hFOB (**D**) and MC3T3-E1 (**E**) cells were cultured in E-plates and, after approx. 24 h, treated with 1 nM–10 μM panobinostat. Cell index was normalized to the time point of treatment. Cell index was determined continuously for additional 80 h. Shown are means ± SD of three independent experiments performed in triplicates. Saos-2 (A), MG63 (B), U2-OS (C), hFOB (D) and MC3T3-E1 (E) cells were cultured in 6-well plates and, after approx. 24 h, treated with 1 nM–100 nM panobinostat. Sub-G1 events were collected and shown are means ± SD of three independent experiments performed in triplicates (right panels).

The time/dose dependent efficacy of panobinostat on osteosarcoma (OS) cell viability was tested using a real-time impedance-based xCELLigence device. Figure 1 shows that 10 nM panobinostat causes a reduction of cell viability after 24 h in Saos-2 (A) and U2-OS (C) cells. MG63 cells (B), apparently more resistant, showed a similar reduction after a longer time of treatment. In Saos-2 cells, 1 nM panobinostat was sufficient to cause a significant reduction of cell viability.

HFOB showed also a significant reduction of cell viability after the treatment with 10 nM panobinostat that could be attributed to their high proliferation rate (Figure 1D).

10 nM panobinostat had no significant toxic effect in MC3T3-E1 mouse osteoblasts used as controls (Figure 1E).

The efficacy of panobinostat was further analysed by flow cytometry to confirm that the reduction of cell viability could be attributed to cell death induction. Here (Figure 1A–1C right panels), the percentage of sub-G1 confined cells, considered apoptotic, increased highly after 24 hours reaching values over 70% after 72 h in all OS cell lines treated with 10 nM panobinostat while untreated controls showed a sub-G1 percentage below 10%. A similar effect was observed in hFOB (Figure 1D right panel), whereas MC3T3 showed a sub-G1 percentage increase only after 72 and 96 h of treatment with 100 nM panobinostat (Figure 1E right panel).

We concluded that concentrations of at least 10 nM panobinostat lead to an induction of cell demise in all osteosarcoma cell lines included in this study. 10 nM panobinostat was considered to be the most efficacious concentration in all three osteosarcoma cell lines and was therefore applied in all further experiments.

### Survivin pathway down-regulation

**Figure 2:**
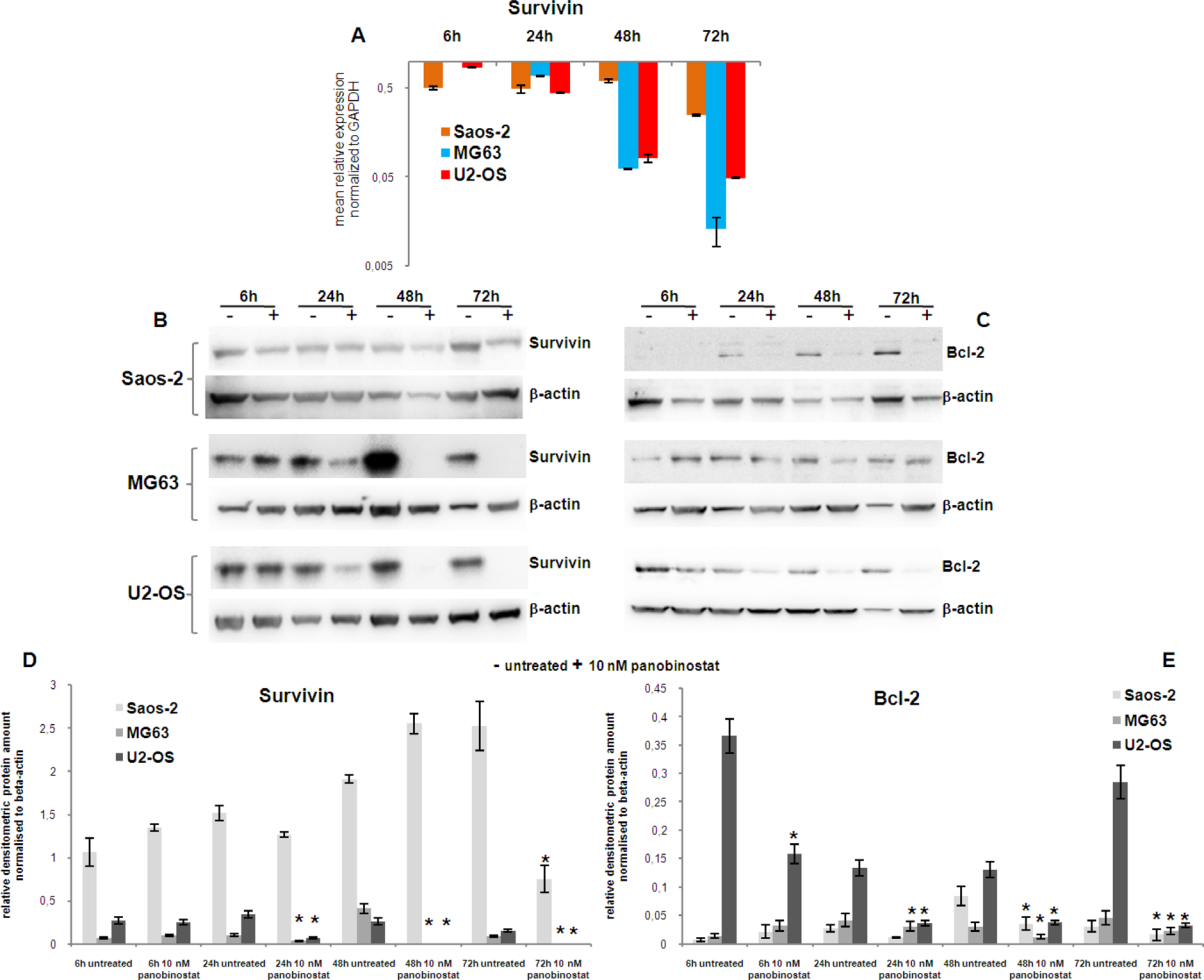
Analysis of survival factors Expression of Survivin transcript (**A**) was determined in all three OS cell lines after 72 h of treatment with 10 nM panobinostat. Shown are means ± SEM of three independent experiments performed in triplicates. The protein level of Survivin (**B**) and Bcl-2 (**C**) was detected in OS cell lines after 72 h of treatment with 10 nM panobinostat. Densitometry results (**D**–**E**) were normalised to β-actin content. ^*^p < 0.05 was regarded as significant: untreated vs panobinostat treated cells.

Survivin is known to be over-expressed in malignant cells; its suppression favours the activation of cell demise mechanisms in cancer [31]. The expression of Survivin and its downstream target Bcl-2 was analysed in osteosarcoma cells. Figure 2A displays that Survivin transcript was significantly down-regulated in Saos-2 after 6 h of treatment with 10 nM panobinostat. This effect was strongly pronounced in MG63 and U2-OS leading to a complete suppression of Survivin transcript after 72 h of treatment. Protein level of Survivin was found, after early treatment (6 h), stable in Saos-2, MG-63 and U2-OS cells (Figure 2B–2D). Prolonged treatment, up to 72 h, caused a progressive down-regulation of Survivin protein in MG-63 and U2-OS cells evidenced by a not detectable Survivin band. Saos-2 cells showed a significant down-regulation of Survivin only after 72 h of treatment (Figure 2B–2D). Interestingly even Saos-2 cells, lacking p53 expression, showed a strong suppression of Survivin transcript, only with a slightly later onset compared to p53 competent MG63 and U2-OS. Earlier suppression in MG63 and U2-OS cells can be explained by p53-dependent suppression induction.

To clarify the effect exerted by Survivin suppression, the protein level of Bcl-2, a downstream target of Survivin and strongly connected to p53 family activity was analysed in OS cells. A strong suppression of Bcl-2 was observed in U2-OS treated with 10 nM panobinostat after 6 h already. MG-63 showed a reduction of Bcl-2 protein level after 24 h of treatment. All three cell lines showed a significant down-regulation of Bcl-2 protein after 48 h of treatment with 10 nM panobinostat (Figure 2C–2E).

These results confirm that panobinostat does reduce the level of the survival factor Survivin and its anti-apoptotic target Bcl-2 in OS cells. Interestingly, even Saos-2 cells, showing a stable protein level of Survivin after long exposure to panobinostat, were characterised by a suppression of Bcl-2. The negative modulation of survival factors mediated by panobinostat could probably increase the efficacy of the cell demise mechanisms induced in OS cells as shown here below.

### P73 and P21 contribute to cell death induction in OS cells

**Figure 3:**
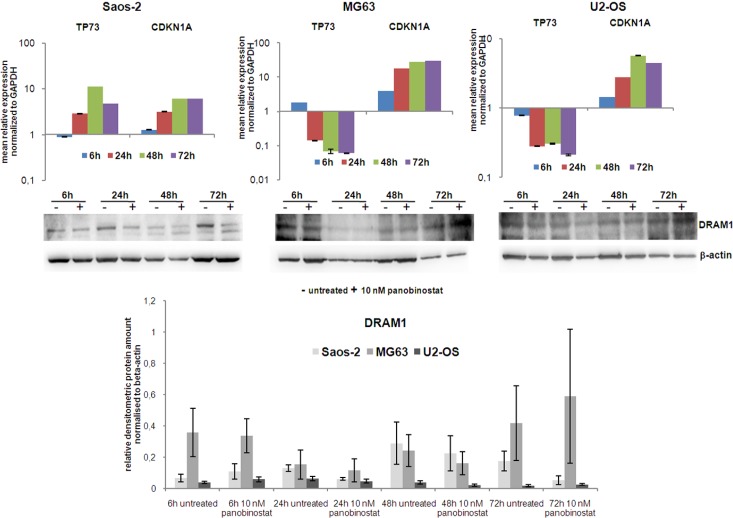
Expression of TP73, CDKN1A and DRAM1 Expression of TP73 and CDKN1A transcripts was determined in all three OS cell lines after 72 h of treatment with 10 nM panobinostat. Shown are means ± SEM of three independent experiments performed in triplicates. DRAM1 protein level (lower panels) was detected in all three OS cell lines. Densitometry results (lowest panel) were normalised to β-actin content.

As we identified a high increase of dying cells after panobinostat treatment, we examined the role of p73 and p21 known as major players in cell cycle control. Therefore, osteosarcoma cell lines were treated with 10 nM panobinostat and gene expression of p73 and p21 was analysed at several time points. Figure 3 (upper panels) shows that expression of p21 was highly increased in all three OS cell lines. Interestingly, p73 transcript (Figure 3 upper panels) and protein expression (Supplementary Figure 1) increased significantly in Saos-2 cells, known to lack p53 expression, after 6 h of treatment. Its level decreased after long time exposure. Here we could confirm that p73, homologue of p53, was induced in p53 deficient cells to overcome the absence of p53.

We then examined protein levels of DRAM1 as a downstream target protein of the p53 transcription factor family, known to be required for autophagy progression. Figure 3 (middle and lower panels) shows an almost stable DRAM1 protein level after treatment with 10 nM panobinostat in OS cells. The shown data indicate that the axis p73 (p53)-DRAM1 could be activated in panobinostat treated osteosarcoma cell lines, thus sustaining the role exerted by DRAM1 during the autophagosome maturation and fusion with lysosomes.

### Induction of autophagy markers

As we have seen earlier, administration of 10 nM panobinostat keeps stable the level of DRAM1 indicating towards an induction of autophagy. We therefore focused on additional autophagy regulating genes.

**Figure 4:**
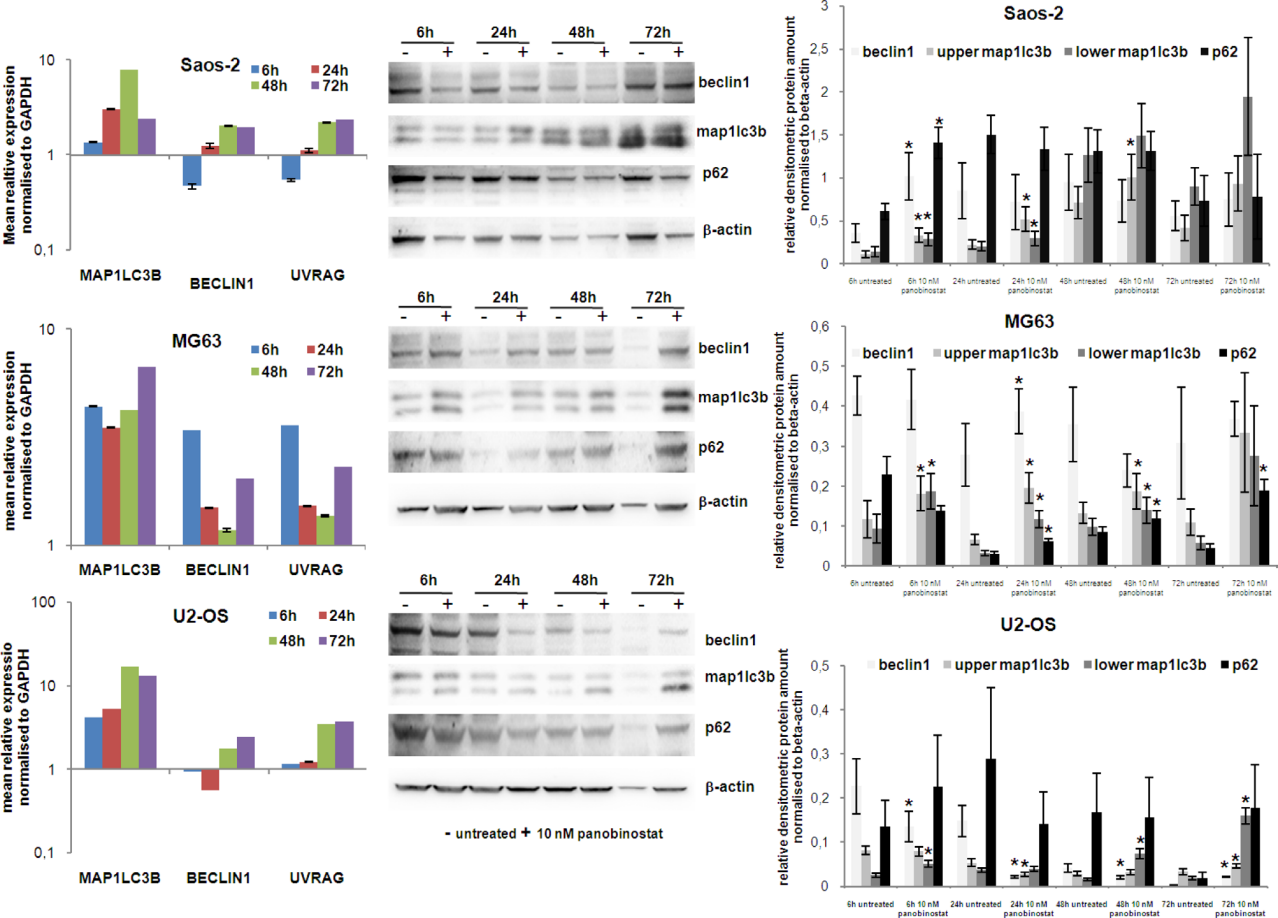
Expression of autophagy markers The graphs on left show the analysis of MAP1LC3B, Beclin1 and UVRAG transcript level in Saos-2, MG63 and U2-OS cells treated for 72 h with 10 nM panobinostat. Shown are means ± SEM of three independent experiments performed in triplicates. Western blot of beclin1, Map1lc3b and p62 was performed in OS cells. Densitometry results were normalised to β-actin content. ^*^p < 0.05 was regarded as significant: untreated vs panobinostat treated cells.

Beclin1 expression is required for the formation of autophagosome vesicles, while UVRAG is responsible for the activation of Beclin1 complex during autophagosome nucleation phase. Both transcripts were induced by 10 nM panobinostat in OS cells (Figure 4 left panels), although induction in Saos-2 and U2-OS cells was detected after 24–48 h, MG63 cells showed an earlier over-expression. Protein levels of Beclin1 were significantly increased in all three cell lines at different treatment time (Figure 4 central and right panels).

The MAP1LC3B gene encodes proteins needed for vesicles assembly during autophagy processing. Expression of this gene was highly elevated in all three cell lines as early as 6 h after treatment with 10 nM panobinostat (Figure 4 left panels). Significant increase of its protein level was observed after 6 h of treatment with panobinostat already. Map1lc3b protein was induced even after longer exposure to 10 nM panobinostat in all OS cells. In particular, the lower molecular weight bands, representing the lipidated active form of Map1lc3b, strongly accumulated confirming an on-going autophagic process (Figure 4 central and right panels).

As autophagy processing relies on building autophagosomes, we analysed levels of the p62 protein. Together with Map1lc3b both proteins are crucial components for the nucleation and elongation of autophagic membrane vesicles. Although at early time point protein levels were significantly increased only in Saos-2 cells, 72 h of treatment caused an increase of p62 protein level also in MG63 and U2-OS cells (Figure 4 central and right panels).

In conclusion, an overall induction of autophagic genes with a consequent over-expression of their protein products could be observed, thus indicating that treatment of osteosarcoma cells with 10 nM panobinostat induces autophagy leading to cell death.

### Involvement of endoplasmic reticulum stress

It is well known that autophagic mechanisms are driven by endoplasmic reticulum stress-related cell death. For this reason, the key factors related to ER stress were also analysed in this model.

**Figure 5:**
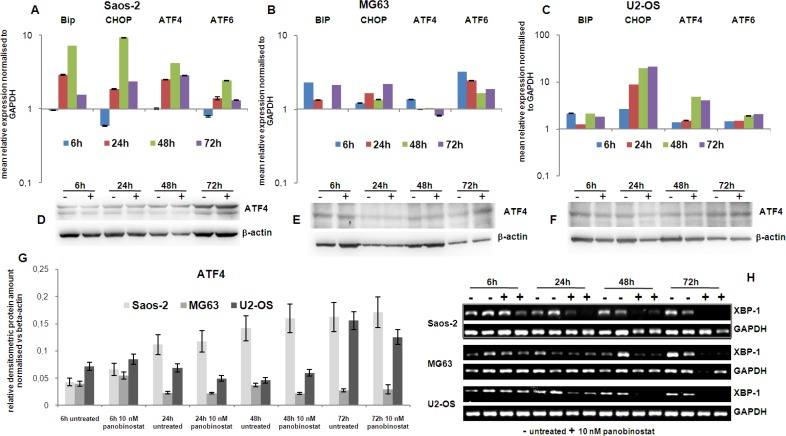
Endoplasmic reticulum stress markers The graphs on the top of the figure (**A**–**C**) show the expression of the transcript of Bip, CHOP, ATF4 and ATF6 genes in OS cells treated for 72 h with 10 nM panobinostat. Shown are means ± SEM of three independent experiments performed in triplicates. In the middle is shown the protein level of ATF4 in Saos-2, MG63 and U2-OS cells (**D**–**F**). Densitometry results were normalised to β-actin content (**G**). Xbp-1 transcript level (**H**) was measured by canonical end point RT-PCR. The band corresponding to the unspliced form totally disappears after treatment with 10 nM panobinostat in all three cell lines. The spliced variant was not even detectable in the untreated cells.

Figure 5A–5C shows the induction of transcript level of BiP, the endoplasmic chaperone, and CHOP, mediating cell death mechanisms, in all three cell lines 24 h after the treatment with panobinostat.

Additionally, the transcription factors ATF4 and ATF6, targeting BiP and CHOP, were up-regulated by 10 nM panobinostat treatment in all three OS cell lines (Figure 5A–5C). In particular, Figure 5 (panels A–C) shows that the expression of these crucial ER-stress mediating transcriptional factors is induced, being visible in all three cell lines after 24 h of treatment with 10 nM panobinostat; ATF4 protein level is stable or slight over-expressed in all three cell lines (Figure 5D–5G).

Xbp-1 is a downstream effector of the protein IRE1α, a key trans-membrane player in the unfolded protein response occurring under ER-stress conditions. Under these circumstances IRE1α is known to splice an intron of xbp-1u (un-spliced) leading to a translation of xbp-1s (spliced) isoform. Figure 5H shows that levels of un-spliced form xbp-1u were reduced time dependently in all three cell lines, assuming a total consumption of xbp-1u in an ER-stress *scenario*. Xbp spliced form was not detectable assuming it was rapidly consumed by the active ER-stress-autophagy process.

We concluded that ER-stress mediated autophagy is responsible for osteosarcoma cells to undergo cell death when treated with 10 nM panobinostat.

### Autophagosomes vesicling

**Figure 6:**
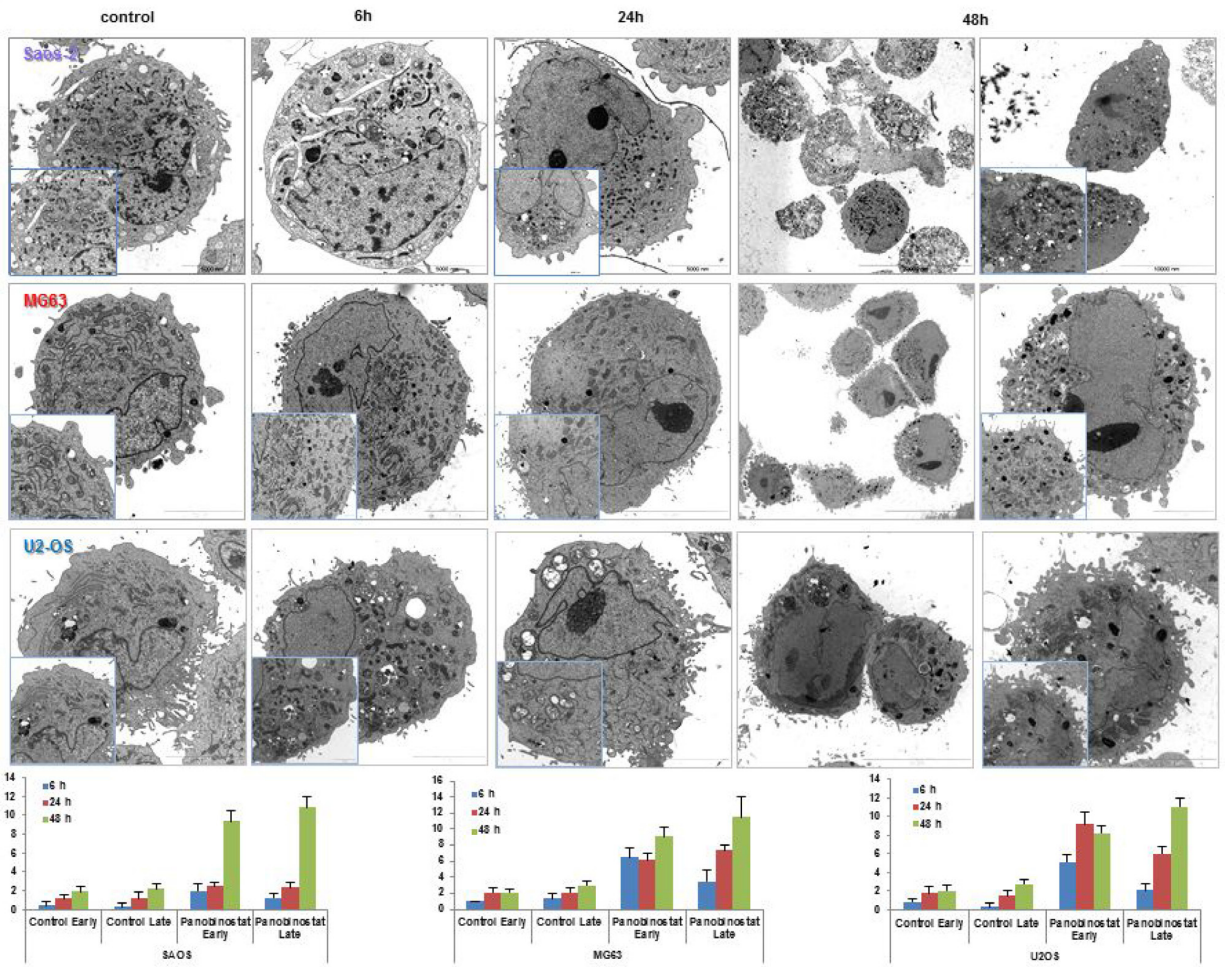
Electron micrographs Transmission electron micrographs showing the disappearance of endoplasmic reticulum and the massive nucleation of autophagosome vesicles in OS cells treated with 10 nM panobinostat. Magnification is 2156 and 35,570 × and scale bars represent 2, 5, 10 and 20 μm. Graphs show the distribution of autophagosomes in OS cells control and after 48 h of treatment with 10 nM panobinostat.

Electron micrograph imaging of Saos-2, MG63 and U2-OS cells treated with 10 nM panobinostat displayed a time dependent increase of fragmented and dissolved endoplasmic reticulum. As shown in Figure 6, endoplasmic reticulum surrounds the cell nucleus. After treatment with 10 nM panobinostat, its structure is completely disorganised and seems to be used for the formation of double membrane autophagic vesicles (Figure 6). After 48 h of treatment, the endoplasmic reticulum appears to dissolve totally and the cells are completely full of autophagic vesicles containing also mitochondrial particles (late autophagosomes). In contrast, untreated controls showed a normal distribution of endoplasmic reticulum and significantly less vesicles.

The number of autophagosome vesicles was quantified based on a previously published method [13, 32, 33]. Statistical analysis was performed, as shown in Table 1. Quantification of early and late autophagosomes revealed a significant time-dependent increase of the vesicles in all panobinostat treated cells, whereby the highest amount of autophagosomes was observed after 48 h of treatment. Panobinostat caused a significant increase of early and late autophagosomes in MG63 and U2-OS cell lines after 6 h, whereas Saos-2 cells showed a significant accumulation of autophagosomes after 48 h of treatment (Figure 6 lower panels). In contrast, untreated controls showed a normal distribution of endoplasmic reticulum and significantly lower number of vesicles compared to panobinostat treated cells.

**Table 1:** Statistical analysis of early and late autophagosomes counting in OS cells control and treated for 48 h with 10 nM panobinostat

	Saos-2			
Mean	Control early	Control late	Panobinostat early	Panobinostat late
**6 h**	0.40	0.30	2.00	1.27
**24 h**	1.15	1.15	2.45	2.36
**48 h**	1.91	2.18	9.33	10.83
**SD**	**Control early**	**Control late**	**Panobinostat early**	**Panobinostat late**
**6 h**	0.49	0.46	0.74	0.45
**24 h**	0.48	0.73	0.50	0.49
**48 h**	0.51	0.57	1.11	1.07
	MG63			
**Mean**	**Control early**	**Control late**	**Panobinostat early**	**Panobinostat late**
**6 h**	1.00	1.33	6.50	3.50
**24 h**	2.11	2.11	6.17	7.33
**48 h**	2.17	3.00	9.13	11.50
**SD**	**Control early**	**Control late**	**Panobinostat early**	**Panobinostat late**
**6 h**	0.00	0.67	1.12	1.50
**24 h**	0.57	0.57	0.90	0.75
**48 h**	0.37	0.58	1.05	2.45
	U2-OS			
**Mean**	**Control early**	**Control late**	**Panobinostat early**	**Panobinostat late**
**6 h**	0.83	0.33	5.13	2.13
**24 h**	1.77	1.54	9.17	6.00
**48 h**	2.00	2.75	8.20	11.00
**SD**	**Control early**	**Control late**	**Panobinostat early**	**Panobinostat late**
**6 h**	0.37	0.47	0.78	0.60
**24 h**	0.80	0.50	1.34	0.82
**48 h**	0.71	0.43	0.75	0.89

In conclusion, OS cells displayed a strong increase of autophagy activity after treatment with 10 nM panobinostat that leads them to cell demise.

### Effects of panobinostat in human and mouse osteoblasts

**Figure 7:**
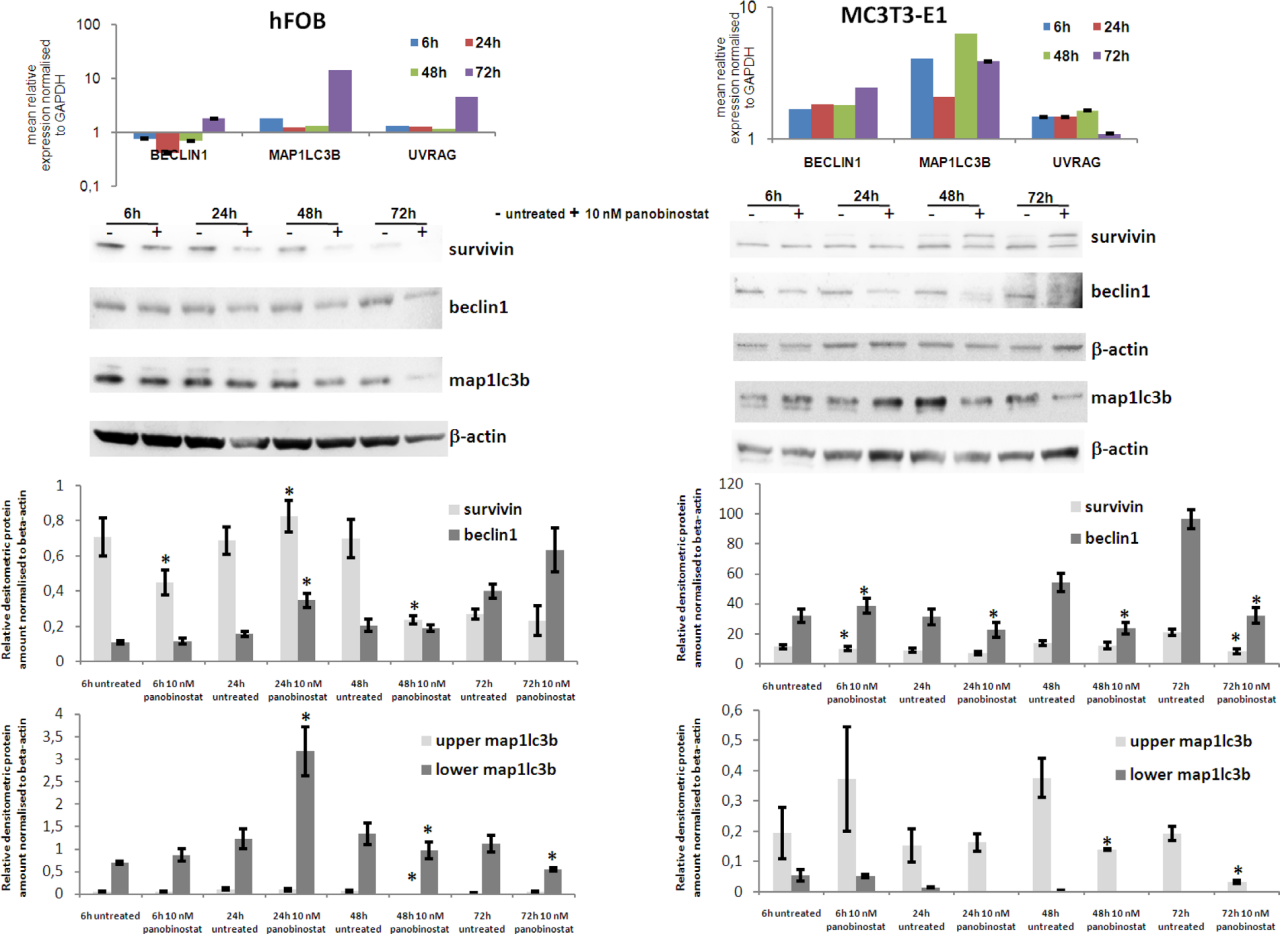
Effects of panobinostat in human and murine osteoblasts Rt-qPCR results of Beclin1, Map1LC3B and UVRAG transcript in human (hFOB) (upper left panel) and murine (MC3T3-E1) (upper right panel) osteoblasts. Shown are means ± SEM of three independent experiments performed in triplicates. Western blot of survivin, beclin1 and map1lc3b in hFOB (middle left panel) and MC3T3-E1 (middle right panel) and further densitometric analysis of the protein level (lower panels). Densitometry results were normalised to β-actin content. ^*^p < 0.05 was regarded as significant: untreated vs panobinostat treated cells.

The effects exerted by 10 nm panobinostat have been analysed in human immortalized osteoblast hFOB 1.1.9 and murine osteoblasts MC3T3-E1. As shown in Figure 7 (upper left panel), the treatment with panobinostat of hFOB cells caused a significant down-regulation of Beclin1 transcript at 24 h, The longer exposure of hFOB to 10 nM panobinostat resulted in a stable expression of Beclin1 transcript. Furthermore, Map1lc3b and UVRAG transcripts are stably expressed up to 48 h of treatment and significantly over-expressed after 72 h of exposure to panobinostat. The protein level of Survivin has also been detected in hFOB. As shown in Figure 7 (middle and lower left panels), the densitometric quantification confirms that survivin is down-regulated at 6 and 24 h of treatment with 10 nM panobinostat; the longer exposure does not cause significant variation of its protein level that is already low in the untreated cells. Beclin1 protein level is generally stable, except for a significant over-expression after 24 h of treatment. Interestingly, the non-lipidated form of map1lc3b is almost no detectable in untreated and exposed cells. The lowest band, corresponding to the active lipidated map1lc3b is stable at 6 h treatment; it increases after 24 h treatment with 10 nM panobinostat and decreases after 48 and 72 h treatment (Figure 7 central and lower left panels).

MC3T3-E1 mouse osteoblasts have been analysed for the expression of autophagy related players. Here, it can be observed that the treatment with 10 nM panobinostat induces a significant over-expression of Map1LC3B transcripts at all time points. Beclin1 is significantly up-regulated after 72 h of treatment. UVRAG transcript is always stable in comparison with untreated cells (Figure 7 upper right panel). Interestingly, Survivin protein level is generally stable and only slightly suppressed after 72 h treatment. Beclin1 protein is slightly up-regulated after 6 h of treatment and its level significantly decreases with longer time exposure. Map1lc3b inactive form (upper band) significantly decreases after 48 and 72 h of treatment with 10 nM panobinostat and its active lipidated form (lower band) is detectable in untreated cells whereas it almost disappears in the cells treated with panobinostat (Figure 7 central and lower right panels).

To summarize, the human and murine osteoblasts, even though having shown a partial significant over-expression of autophagy markers transcripts, are characterized by a stable and/or down-regulated protein level of survivin and autophagy markers. This could suggest a possible suppression of autophagy mechanism in normal cells of the bone marrow.

## DISCUSSION

Osteosarcoma remains to be associated with a poor overall prognosis and therapeutic advances are urgently required. Improving our understanding of the mechanism promoting cell demise in malignant bone cancer cells appears to be promising.

Recent research focused on the potential of deacetylase inhibitors for their wide range of activity that determine cell differentiation and cell demise with different modalities in blood and solid malignancies, thus weakening tumour aggressiveness and metastatic potential. In particular, it has been observed that the combination of deacetylase inhibitors like SAHA and heavy ion radiotherapy is able to block OS xenograft tumour growth in mice [34]. Furthermore, SAHA synergistically enhances the cytotoxic effect of etoposide in Ewing sarcoma [35]. Interestingly, the use of bone cement loaded with histone deacetylase inhibitors has shown to be associated with a lower rate of metastasis and a better survival in patients affected by osteosarcoma and chondrosarcoma [36]. Panobinostat has also shown to cause differentiation at low dosage leading to reduction of tumour growth in osteosarcoma xenografts [37].

Up to now, the studies focused only on the potential of these compounds to reduce the aggressiveness of osteosarcoma cells to metastasize and invade the surrounding tissues.

Here, we showed that low concentrations of panobinostat do not only reduce cell survival but also initiate alternative cell death mechanisms. There remains an ongoing debate regarding the exact interaction between apoptosis and autophagy during phases of cell stress. Recent studies showed that autophagy can account not only for cell protection but also lead to cell death if it is not restrained [8].

Recently, we have been able to show similar ways of action caused by panobinostat in liver cancer [12] and we further clarified that panobinostat acts by inducing ER stress and autophagy mediated cell death [13, 15]. Here we could observe that OS cells are even more sensitive to panobinostat as well as thyroid cancer cells [38, 39]. Lower concentration of panobinostat potently induced a reduction of survival rate and an increase of cell death rate *via* ER-stress mediated autophagy.

Survivin is a survival factor assuming an oncogenic role in cancer due to its abnormal over-expression conferring survival resistance to cancer cells [31]. It has been shown that OS is characterized by high amounts of this protein conferring to cells a peculiar resistance to die and for this reason could represent a valid biomarker for OS patients [40]. The current strategy is focusing on finding potent Survivin inhibitors that could improve the therapy in combination with canonical chemotherapeutics as shown for YM155 [41]. Survivin and its downstream target Bcl-2 were significantly suppressed in osteosarcoma cells after a short time of treatment with low concentrations of panobinostat. Nonetheless a strong over-expression of the transcript of cyclin dependent kinases inhibitor p21^cip1/waf1^ was observed. Thus, supporting that panobinostat could represent a valid compound for reducing the resistance of OS to chemotherapy. Based on our previous studies showing that panobinostat promoted alternative cell death mechanisms [12, 13, 15], this study aimed to analyse the status of ER-stress and autophagy in OS cells. DRAM1, an autophagic marker transcribed by p53, is able to trigger autophagy and/or apoptosis in normal and cancer cells [42, 43]. In our study, DRAM1 was stable thus suggesting its active role in cell death mechanisms in OS cells treated with panobinostat.

The autophagic markers Beclin1, MAP1LC3B and p62 were affected by panobinostat treatment. It could be observed that these proteins were stable or over-expressed at different time points depending on the OS cell line. After long term treatment, all analysed markers strongly accumulated matching the observed massive autophagosome vesiculation.

Treatment with panobinostat caused a significant over-expression of ER stress markers BiP, ATF4, ATF6 and CHOP. Furthermore, the disappearance of the unspliced Xbp variant, a downstream target of ER stress signalling pathway, could sustain the activation of cell death and mediate autophagy by transcriptional activation of Beclin1 as previously shown [44].

Interestingly, the endoplasmic reticulum was profoundly damaged and disappeared after treatment with panobinostat. Instead, the amount of autophagosomes significantly increased supported by a massive reduction of autophagosome membrane proteins and the disappearance of endoplasmic reticulum from the peri-nuclear subcellular area. The mechanisms induced in OS cells by panobinostat did not differ from the ones induced in liver cancer [13, 15] but were stronger and promoted by a far lower dose than previously used in other studies. Additionally, this study showed that the mechanism activated by panobinostat was a specific mode of action executed in OS cells. Human and murine osteoblasts treated with the histone deacetylase inhibitor showed a different expression profile of autophagy related markers and survivin. Those cells were characterized by a nearly stable survivin protein and, despite the over-expression of Map1lc3b transcript, its protein level and that one of beclin1 was always down-regulated. The suppression of autophagy mechanism could support the survival of osteoblasts to the treatment with panobinostat.

Therefore, panobinostat could represent a potential substance for the treatment of malignant primary bone tumours due to its property to suppress survival mechanisms and potentiate the death signalling in those cells only.

Modulation of cell death mechanisms by the use of epigenetic modulators could overcome the resistance of malignant primary bone tumours to canonical chemotherapeutics and ameliorate the survival rate of affected patients [45]. Further studies dissecting the possible effect on miRNAs are strongly needed as it has been shown that the small non-coding RNAs play a key role during bone tumorigenesis [46]. Panobinostat could probably alter the expression of miRNAs in favour of tumour-suppressor miRNAs in OS as it has been proven before in other solid tumours [11, 14].

## MATERIALS AND METHODS

### Cell lines

Osteosarcoma cell lines Saos-2 (ACC243, Leibniz Institute DSMZ, German Collection of Microorganisms and Cell Culture), MG63 (CRL-1427, ATCC, Manassas, USA) and U2-OS (kindly gift from Toh Weng Tan, Institute of Experimental Orthopaedics, Philipps University of Marburg) were grown in DMEM (Gibco, Paisley, UK) and MC3T3-E1 (ACC 210, DSMZ, Germany) mouse embryo osteoblasts were grown in MEM Alpha Medium (Gibco) both supplemented with 10% fetal bovine serum, 2 mM L-Glutamine, penicillin (107 U/l) and streptomycin (10 mg/l) (Biochrom AG, Berlin Germany) at 37°C in a humidified atmosphere containing 5% CO_2_. hFOB 1.19 (CRL-11372, ATCC) were grown with 1:1 mixture of Ham's F12 Medium Dulbecco's Modified Eagle's Medium, with 2.5 mM L-glutamine, 0.3 mg/ml G418 and fetal bovine serum to a final concentration of 10% at 34°C to keep them undifferentiated. The temperature was raised up (39–40°C) to obtain a differentiated population that was also included in the study. Panobinostat, a kindly gift from Novartis AG, Basel Switzerland, was dissolved as previously described [12].

### Impedance-based real-time cell analysis (RTCA)

The xCELLigence RTCA SP system (OLS Omni Life Science, Bremen Germany) was used for real-time and time-dependent analysis of the cellular response of Saos-2, MG63, U2-OS, hFOB cells and mouse embryo fibroblasts following the incubation with 1 to 100 μM panobinostat. In brief, 5 × 10^3^ Saos-2, MG63, U2-OS, hFOB and MC3T3-E1 cells per well were seeded in triplicate in 150 μl complete growth medium in a 96-well E-plate (OLS). After approx. 24 h, cells were washed and incubated in complete growth medium with or without 1 nM-10 μM panobinostat. Cell viability was then measured on cell impedance based detection that results in variation of current voltage related to cell attachment/detachment and morphological changes on the plate's electrodes. Continuous acquisition was performed for 120 h. Data were analysed using the RTCA Software V1.2.1 (OLS) [11, 47, 48].

### Analysis of cell death

Flow cytometry was employed for the quantification of cell death in treated cell lines after staining with propidium iodide as described previously [12, 15]. Analysis of labelled nuclei was performed on Attune acoustic flow cytometer with Attune Cytometer Software 2.1.0 (Life Technologies GmbH, Darmstadt, Germany).

The percentage of apoptotic cells was determined by measuring the fraction of nuclei with a sub-diploid DNA content. Ten thousand events were collected for each analysed sample.

### RT-qPCR

Total RNA was isolated with the RNeasy Mini Kit (74106, Qiagen, Hilden Germany) according to the manufacturer's protocol. RNA concentration was measured photometrically and the extinction coefficients E260/E230 and E260/E280 were used as purity control of the samples. The mRNA was reverse transcribed with iScript™ cDNA Synthesis Kit (170-8891, BIORAD, Munich Germany) and amplified with the SsoFast^™^ EvaGreen^®^ Supermix (BIORAD, 172-5200) on a CFX96 Real Time PCR Detection System (BIORAD). GAPDH (QT01192646), TP73 (QT00030240), CDKN1A (QT00062090), BiP (HSPA5, QT00096404), CHOP (DDIT3, QT00082278), ATF4 (QT01678523), ATF6 (QT00083370), Survivin (BIRC5, QT00081186), MAP1LC3B (QT00055069), BECN1 (QT00004221) and UVRAG (QT00034328) primers were purchased by Qiagen. Results were analysed with the CFX Manager v2.0 and Rest 2008 software and normalized to GAPDH mRNA content for each sample.

### Semi quantitative RT-PCR

For semi-quantitative PCR, the Ready Mix Taq PCR Kit (Sigma-Aldrich, Munich Germany) was used. The oligonucleotides 5′-CCTTGTAGTTGAGAACCAGG-3′ and 5′-GGGGCTTGGTATATATGTGG-3′ (Eurofins MWG Operon, Ebersberg, Germany) were used for amplification of the X-box binding protein 1 (XBP-1) transcript fragments. PCR products were resolved on two different 2% agarose gels, stained with Sybersafe DNA gel stain (Life Technologies) and visualised under ultraviolet illumination using Fusion image capture (PEQLAB Biotechnologie GmbH, Erlangen, Germany). Glyceraldehyde-3-phosphate dehydrogenase (GAPDH) was amplified as internal control.

### Protein extraction and Western blot analysis

Whole cell lysates were further processed by SDS-Page followed by Western blotting, as previously described [15]. Immunodetection with primary antibodies against human Survivin (AF886, R&D Systems, Wiesbaden-Nordenstadt, Germany), Bcl-2 (610539, BD Transduction Laboratories) Beclin1 (ab114071), Map1lc3b (ab51520) and Sqstm1 (ab96706) (AbCam), DRAM1 (PRS4035 Sigma-Aldrich Saint Louis MO USA), ATF4 (ab50546, AbCam) and β-actin (A5441) (Sigma-Aldrich) was performed. Bound secondary HRP-conjugated anti-mouse (A9917) and anti-rabbit (A0545) antibodies (Sigma-Aldrich) were detected by incubating the immunoblots with Super Signal West Pico Chemiluminescent Substrate (#34077, Pierce, Thermo Fisher Scientific, Darmstadt Germany). The luminescent reactivity was then measured using Fusion image capture and further quantified with Bio1D analysis system (PEQLAB Biotechnologie GmbH). The blotted nitrocellulose membranes (Amersham Protran Premium 0.2 μm NC Cat. N. 10600009 Ge Healthcare Life Science, Freiburg Germany) were up to four times stripped with Stripping Buffer (Restore Plus Western Blot Stripping Buffer Cat. N. 46430 Thermo Scientific) as suggested by the company and reprobed in order to detect other proteins. At last, anti-β-actin was used to control equal loading and protein quality.

### Sample processing for transmission electron microscopy (T.E.M.)

Saos-2, MG63 and U2-OS cells were treated with panobinostat for 48 h. Cells were processed as previously described [13, 15, 49]. Additionally, early and late autophagosomes were quantified as published recently [13].

### Statistical analysis

Statistical analysis was performed using SPSS 15.0.1 for Windows (SPSS Inc, Chicago, IL). The *t* test or ANOVA with Bonferroni corrections (Bonferroni post hoc test) were used to test for differences between two or more groups of samples, respectively. *P* < 0.01 and *P* < 0.05 were regarded as significant.

## SUPPLEMENTARY MATERIALS FIGURE


