# Supplementary material for: Panobinostat mediated cell death: a novel therapeutic approach for osteosarcoma
Source: Oncotarget. 2018 Aug 31;9(68):32997–3010. doi: 10.18632/oncotarget.26038 (PMC6152475; doi:10.18632/oncotarget.26038)
Supplement: Supplementary file 1 [file oncotarget-09-32997-s001.pdf]

Panobinostat mediated cell death: a novel therapeutic approach for osteosarcoma

SUPPLEMENTARY MATERIALS

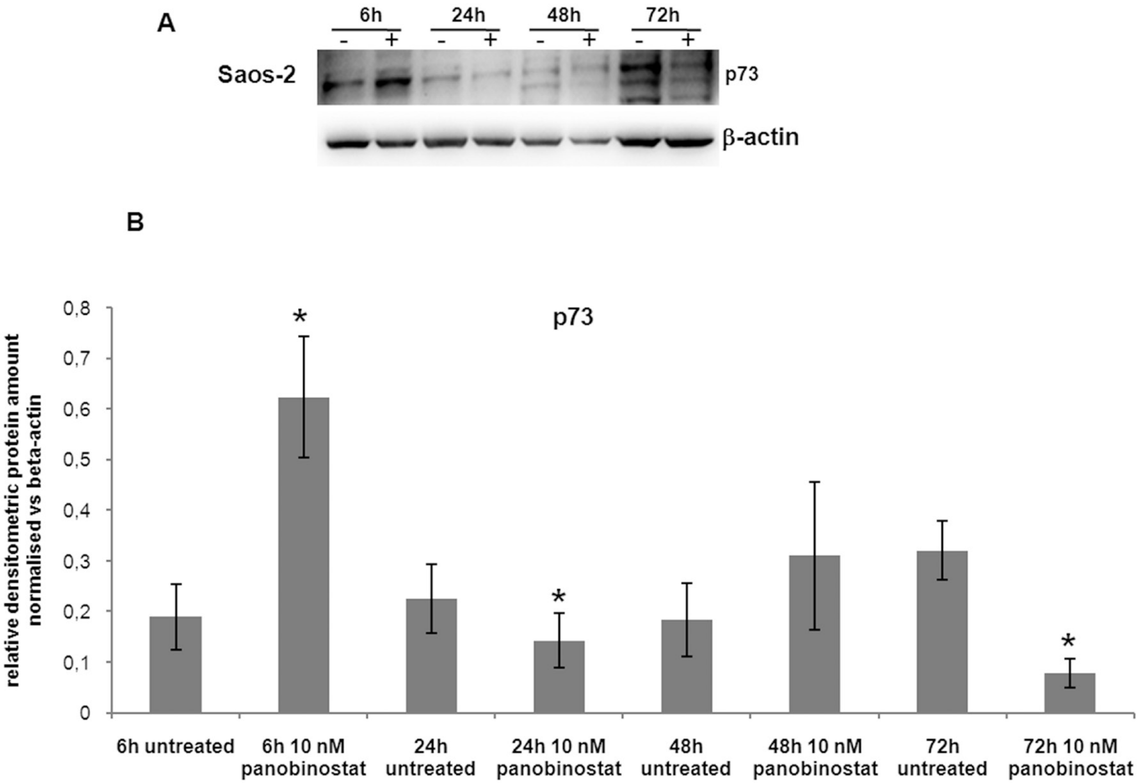

Supplementary Figure 1: Western blot in panobinostat treated Saos-2 cells. TP73 protein level was detected in Saos-2 cell line. Densitometry results were normalized to β-actin content. $^{*}p < 0.05$ was regarded as significant.
